# Publisher’s Note: Continued Publication of *Current Issues in Molecular Biology* by MDPI

**DOI:** 10.3390/cimb43010001

**Published:** 2021-03-18

**Authors:** Franck Vazquez, Shu-Kun Lin

**Affiliations:** MDPI, St. Alban-Anlage 66, CH-4052 Basel, Switzerland; lin@mdpi.com

*Current Issues in Molecular Biology* (ISSN 1467-3045) was launched in 1999 and has published international and multidisciplinary articles on all aspects of molecular biology spanning from basic mechanisms to applications in fields primarily, but not exclusively, relevant to microbiology and virology. *Current Issues in Molecular Biology*, published by Caister Academic Press [1] over the past 23 years, has been the home for 388 peer-reviewed articles across 42 volumes. As of 5 March 2021, *Current Issues in Molecular Biology* will be published by MDPI [2].

We are delighted to take over the publication of *Current Issues in Molecular Biology,* as it excellently complements the portfolio of MDPI journals [3], provides a multidisciplinary addition to our existing journals in this field, such as the *International Journal of Molecular Sciences* [4] and strengthens our service to the relevant scientific communities. 

*Current Issues in Molecular Biology* will publish papers which provide fundamental mechanistic and functional insights into molecular biology, in both eukaryotes and prokaryotes. MDPI will publish three issues in 2021, and four quarterly issues as of 2022. 

We look forward to publishing your work in *Current Issues in Molecular Biology*!
